# Burnout and Associated Psychological Problems Among Teachers and the Impact of the Wellness4Teachers Supportive Text Messaging Program: Protocol for a Cross-sectional and Program Evaluation Study

**DOI:** 10.2196/37934

**Published:** 2022-07-14

**Authors:** Belinda Agyapong, Yifeng Wei, Raquel da Luz Dias, Vincent Israel Opoku Agyapong

**Affiliations:** 1 Department of Psychiatry Faculty of Medicine and Dentistry University of Alberta Edmonton, AB Canada; 2 Department of Psychiatry Faculty of Medicine Dalhousie University Halifax, NS Canada; 3 Nova Scotia Health Authority Halifax, NS Canada

**Keywords:** burnout, stress, Wellness4Teachers, anxiety, depression, e-mental health, teachers, support, text message, mental health, SMS, high school, elementary school, prevalence, psychological intervention, school

## Abstract

**Background:**

Stress, burnout, anxiety, and depression continue to be a problem among teachers worldwide. It is not presently known what the prevalence and correlates for these psychological problems are among teachers in Alberta and Nova Scotia. It is also not known if a supportive text message program (Wellness4Teachers) would be effective in reducing stress, burnout, anxiety, or depression symptoms among teachers.

**Objective:**

The goal of this study is to evaluate the prevalence and correlates of stress, burnout, symptoms of anxiety, depression, and low resilience among elementary and high school teachers in Alberta and Nova Scotia, Canada. It also aims to determine if daily supportive text messages can help reduce the prevalence of these psychological problems in teachers.

**Methods:**

This is a cross-sessional mixed methods study with data to be collected from subscribers of Wellness4Teachers using a web-based survey at baseline (onset of text messaging), 6 weeks, the program’s midpoint (3 months), and end point (6 months). Teachers can subscribe to the Wellness4Teachers program by texting the keyword “TeachWell” to the program phone number. Outcome measures will be assessed using standardized rating scales and key informant interviews. Data will be analyzed with descriptive and inferential statistics using SPSS and thematic analysis using NVivo.

**Results:**

The results of this study are expected 24 months after program launch. It is expected that the prevalence of stress, burnout, anxiety, depression, and low resilience among teachers in Alberta and Nova Scotia would be comparable to those reported in other jurisdictions. It is also expected that factors such as gender, number of years teaching, grade of teaching, and school type (elementary vs high school) will have an association with burnout and other psychological disorders among teachers. Furthermore, it is expected that Wellness4Teachers will reduce the prevalence and severity of psychological problems in teachers, and subscriber satisfaction will be high.

**Conclusions:**

The Wellness4Teachers project will provide key information regarding prevalence and correlates of common mental health conditions in teachers in Alberta and Nova Scotia, as well as the impact of daily supportive text messages on these mental health parameters. Information from this study will be useful for informing policy and decision-making concerning psychological interventions for schoolteachers.

## Introduction

### Background

Educators and the public alike see stress and burnout as a distinct problem of the teaching profession [1,2]. Burnout is defined as a state of emotional, mental, and physical exhaustion resulting from prolonged and lengthy stress at work [3,4], as well as a response to chronic emotional and interpersonal stressors. Burnout is defined by the 3 dimensions of emotional exhaustion, cynicism, and inefficacy [5]. Emotional exhaustion represents emotional depletion and loss of energy; depersonalization or cynicism is the interpersonal dimension of burnout and refers to a negative, callous, or excessively detached response to other people. Reduced accomplishment describes the self-evaluation dimension of burnout and refers to feelings of incompetence and a lack of achievement and productivity at work [5,6]. In a cross-sectional study, 33.3% of teachers reported high burnout while 27.6% were at risk for having moderate burnout [7]. Similarly, 34.9% of teachers indicated they might be threatened by burnout syndrome [8].

Literature shows that teachers experience considerable stress in the workplace resulting in higher burnout rates, which poses a health risk [7,9]. A recent study by Li et al [10] reported that 53.2 % of teachers identified work as a source of long-term stress, leading to burnout. The results of a transversal study conducted in Tunisia reported that most teachers (66.4%) acknowledged being stressed at work, and burnout syndrome was found in 21% of those teachers [3]. A systematic review indicated that burnout was an important predictor of both physical and psychological consequences, including insomnia, depressive symptoms, and mortality below the age of 45 years; hospitalization for mental disorders; and psychological ill-health symptoms [11]. Another study reported that physical illnesses are more common among individuals with burnout compared with those without (64% vs 54%; *P*<.001) with increased prevalence of diseases associated with severity of burnout [12]. A study finding also showed that high burnout was associated with a high level of emotional exhaustion, low personal accomplishment, and depersonalization, while low burnout was linked to high personal accomplishment [7]. The professional outcomes of burnout include job dissatisfaction and absenteeism [11]. Absence due to sickness was more prevalent among employees with burnout compared with those without burnout [13]. The level of satisfaction is a significant factor that impacts the mental and physical health of teachers as well as other workers. Those with low job satisfaction are more susceptible to experiencing burnout, high anxiety levels, depression, and low self-esteem [7,14]. A study also showed that participants who reported high social anxiety levels reported high burnout levels as well [15]. A survey conducted in Canada and the United States confirmed that workplace improvements could prevent adverse sequelae, improve health outcomes, and reduce health care expenditures [16]. Data on the prevalence of burnout and other psychological problems among teachers in Canada are limited in the literature. It has been suggested that this is because North American jurisdictions have been hesitant to recognize burnout as a clinical diagnosis, partly due to concerns about increasing requests for disability coverage [6].

The relationship between burnout and health exhibits complicated pattern in the sense that poor health contributes to burnout, and burnout contributes to poor health [17]. Burnout is a risk factor for poor physical and mental well-being, and it may adversely affect health [11,18]. There is increasing evidence that burnout as a stress response represents a risk factor not only for depression but also for cardiovascular and other somatic diseases [19]. Burnout is occupational-specific dysphoria, which is different from depression—a general mental illness [5]. Burnout is regarded as a stress-related state, and the rates of clinical depression increase with the severity of burnout [19]. Burnout is also job related and dependent on the situation or condition, while depression is more general and context‐free [6]. Cross-sectional studies showed that there is a relationship between burnout and depression with greater risk of major depressive disorder (MDD) when burnout is severe [12]. Teachers with MDD also had higher levels of perceived stress, anxiety, disorder, and lower quality of life [20]; moreover, poor workplace environment was a factor associated with both increased anxiety and depressive symptoms [21]. The published results of a study reported a high prevalence of depression (49.1%) among teachers [22] as well as a relatively high prevalence of anxiety 68.0% [23].

In a longitudinal study, individual teachers who experienced an increase in the states of burnout had an increase in depression in comparison to those with decrease in burnout, which corresponded to less depression [7,24]. This was also observed in a study where 86% of the teachers who identified as burned-out met the criteria for a provisional diagnosis of depression [25].

The results of a study in Quebec, Canada found that the proportion of teachers who reported a high level of psychological distress was twice as high (40%) than that reported for a Quebec-wide sample (20%) [26], an indication that teachers in Canada are also prone to stress. The ability to be able to cope under stress and pressure is an important factor to reduce burnout. Self-efficacy is viewed as a significant personal resource associated with coping with stress, and teacher’s self-efficacy was positively correlated with general self-efficacy but negatively with job burnout [27]. According to psychologists, resilience is the process of adapting well in the face of adversity, trauma, tragedy, threats, or significant sources of stress including workplace stressors [28]. Low resilience is predicted to reduce the individual’s ability to cope with stress and therefore will lead to increased levels of burnout. Increased levels of job support, however, were a protective factor against emotional exhaustion [29]. A study’s results also indicated that the prevalence of resilience was exclusively predicted by factors including the participant’s level of trauma exposure, social support, and recent and past life stressors [30].

Elementary and high school teachers must constantly deal with students’ discipline issues while at the same time ensuring the timely delivery of the curriculum. Teachers must also deal with their own personal and family day-to-day stressors while helping to support students under their care. This leads to heightened levels of occupational stress and amplified risk of mental disorders [1]. The stress of the job sometimes takes a toll on the teachers, giving rise to burnout, increased anxiety and depression, and sometimes reduced resilience [5,17,31]. Meditation is effective in improving resilience, psychological distress, fatigue, and burnout. Thus, teachers may benefit from in-school wellness programs that incorporate mindfulness and meditation [31]. Additionally, the quest for professional fulfillment is more challenging in schools with high discipline issues and poor classroom settings. Furthermore, in the last 2 years, the global pandemic has led to changes in the school system, including intermittent web-based learning, closure of schools, and cancellation of provincial exams, thus creating major uncertainties for both students and teachers. It is currently unknown if the additional stressors experienced from the pandemic impacts the levels of stress, anxiety, depression, burnout, and resilience in teachers in elementary and high schools. Given the aforementioned, teachers may benefit from in-school intervention and wellness programs to alleviate their stress and burnout. Meditation techniques such as mindfulness have been suggested to help teachers cope with stress and burnout. Mindfulness is the practice of bringing awareness to the here and now using a variety of methods [32], and it has been suggested as beneficial in coping with job-related stress and burnout in the teaching profession [33,34]. Mindfulness can also improve the interpersonal faculties of teachers’ sense of efficacy and perceived stress [33], as well as reducing depression [34]. However, the nature of these programs requires focusing and concentration, and this can be challenging to pursue in the busyness of the school environment and especially when teachers are stressed or burnt-out. Thus, the teachers may not be in the position to complete these programs. As mentioned, these programs can be time-consuming and may require teachers to consciously set time apart to participate. In addition, mindfulness may not be easily accessible and scalable in all schools. Furthermore, mindfulness may not be suitable or convenient for some teachers. An innovative way to offer intervention to teachers is through mobile text technology.

Mobile text technology is a unique and innovative way that offers a convenient, low-cost, and easily accessible way of delivering psychological interventions to the general public with mental health problems [35]. Supportive text messages can be used to supplement mental health therapy and hence indirectly reduce waitlist and the number of days required to attend group-based or face-to-face therapy programs that span months and require a lot of human resources for their delivery. Text messages have been effectively used to support the mental health of the general Albertan population and was effective in reducing stress, anxiety, and depression [36,37]. Supportive text messaging has also been used to reduce depression and increase abstinence duration in alcohol use disorder [38,39]. It is currently unknown whether supportive text messages will be helpful in reducing stress, anxiety, and depression while improving resilience among elementary and high school teachers. Given the generally high psychological burden among teachers and the evidence of effectiveness of supportive text message interventions in the literature, we propose to use Wellness4Teachers, a supportive text message program to help reduce stress, burnout, anxiety, and depression and to improve resilience among teachers.

### Wellness4Teachers Program

Wellness4Teachers is a self-subscription daily supportive text message intervention program designed to address stress, burnout, anxiety, and depression, and to build resilience and improve professional satisfaction in teachers. The Wellness4Teachers program is powered by the ResilienceNHope web-based application [40] and provided by the Global Psychological eHealth Foundation [41]. ResilienceNHope is an evidence-informed e–mental health program that incorporates cognitive behavioral therapy based on daily supportive messages (mobile text or email), weekly mental health literacy information, web-based mental health self-assessments, and other mental health resources to help address part of the mental health literacy and the psychological treatment gap for individuals and communities globally. The Wellness4Teachers application will deliver one-way (noninteractive) psychological intervention messages to mobile phones. Subscribers will be made aware of the noninteractive nature of the supportive messaging program through the welcome and introductory message they receive upon subscribing to the program. They will also be offered the phone number of the mental health crisis service for their province or region to call if they are in crisis. The daily supportive messages delivered through the Wellness4Teachers program were crafted by psychiatrists, mental health therapists, and psychologists based on the cognitive behavioral therapy principles. The messages were further reviewed by the lead author, who is an education specialist, before they were built into the Wellness4Teachers program. Different messages will be received daily from a bank of messages. Examples of the text messages are as follows:

Deep breathing is a skill. You may need to practice it often and for more than 5 minutes to feel calmer. When you are feeling stressed or tense, shrug your shoulders up to your ears, hold for 5 seconds, release and repeat 2 more times. https://www.youtube.com/watch?v=cOOd-wlMMRg.

As a teacher, it is very important for your well-being to spend time talking, laughing, and sharing with your colleagues. If you feel overwhelmed, take time to do something kind for yourself such as a cup of tea, or nature walk.

Visualize yourself coping with the current challenging student behavior or workload. See yourself confidently facing these challenges. You can do it! No matter the challenges.

Teachers in both Alberta and Nova Scotia can subscribe to the Wellness4Teachers program by texting “TeachWell” to a designated phone number to be automatically registered to receive supportive text messages. Teachers will receive supportive text messages at no cost to themselves or their institutions and will not receive any reimbursement or incentives for participating. Wellness4Teachers supportive text messages will be delivered to subscriber cell phones at 12 PM Mountain Standard Time in Alberta and 9 AM Atlantic Standard Time in Nova Scotia each day, and subscribers will receive the daily messages for 6 months. Based on a 10% dropout rate recorded for the Text4Hope program [36,37], the dropout rate expected for the Wellness4Teachers program is less than 15% [42].

The effectiveness of the ResilienceNHope suite of programs have been evaluated and established through several randomized controlled clinical trials and evaluations of population level programs. In a randomized controlled trial (RCT) in Fort McMurray, Alberta, Canada, involving 73 patients diagnosed with MDD, the intervention group (n=35, 48%) received twice-daily supportive text messages for 3 months (intervention group) as part of their outpatient treatment, while the control group (n=38, 52%) received a single thank-you message (control group) every fortnight (20.8, SD 11.7 vs 24.9, SD 11.5, respectively; *F*_1,60_=4.83; *P*=.03, η_p_^2^=0.07). The mean difference in the Beck Depression Inventory (BDI) score change was significant with an effect size (Cohen *d*) of 0.67 [43]. In an earlier RCT in Dublin, Ireland, 54 patients with MDD and comorbid alcohol use disorder were also randomized to receive either twice-daily supportive text messages (intervention group) or a thank-you text message (control group) for 3 months. After adjusting for baseline scores, there was a statistically significant difference in the 3-month BDI-II scores between the intervention and control groups (8.5, SD 8.0 vs 16.7, SD 10.3, respectively; *F*_1,49_=9.54; *P*=.003, η_p_^2^=0.17. The mean difference in change BDI-II scores was –7.9 (95% CI –13.06 to –2.76; Cohen *d*=0.85) [39]. Subscribers of Text4Hope (launched in Alberta during the COVID-19 pandemic [44]), who had been enrolled for 6 weeks (intervention group) had a significantly lower prevalence of moderate-to-high stress (78.8% vs 88.0%); moderate-to-high anxiety symptoms (31.4% vs 46.5%); and moderate-to-high depression symptoms (36.8% vs 52.1%), suicidal ideation (16.9% vs 26.6%), and disturbed sleep (76.9% vs 85.1%) compared to new subscribers, respectively, during the same time period (control group) [45]. Furthermore, there were statistically significant reductions in both the prevalence and mean scores on standardized measures for stress, anxiety, and depression at 6 weeks and 3 months for subscribers to the Text4Hope program [36,37].

### Study Aims

One goal of this study is to evaluate the prevalence and correlates of burnout, probable mental health disorders, and low resilience among elementary and high school teachers in Alberta and Nova Scotia, Canada. Another goal of this study is to determine if daily supportive text messages can help reduce the prevalence of burnout, stress, symptoms of anxiety, and depression, and improve resilience among elementary and high school teachers (Textbox 1).

**Study objectives.**

**Main objectives**

- To determine the prevalence and correlates of burnout, moderate to high stress, likely generalized anxiety disorder, likely major depressive disorder, and low resilience among elementary and high school teachers in Alberta and Nova Scotia
- To determine if the daily supportive text messaging program, Wellness4Teachers, can reduce the prevalence and severity of stress, burnout, anxiety, and depression, and to improve resilience among elementary and high school teachers in Alberta and Nova Scotia
- To assess Wellness4Teachers program subscriber experience and satisfaction with the daily supportive text messaging program

## Methods

### Study Design

This study will use a mixed-methods quantitative and qualitative cross-sectional survey design. Quantitative data will be collected using web-based–administered questionnaires through the University of Alberta REDCap platform [46], a secure web application for building and managing web-based surveys and databases. Qualitative data will be collected through key informant interviews.

The web-based questionnaires will be designed to collect demographic, professional, and clinical variables including stress, burnout, anxiety, depression, and resilience. Follow-up surveys will also include subscriber experience and satisfaction questions. Teachers in Alberta and Nova Scotia will be invited to subscribe to the Wellness4Teachers program through an advertisement organized in collaboration with Alberta Teachers Association, the Alberta School Boards Association, the Nova Scotia School Boards Association, and the Nova Scotia Teachers Union. The web-based surveys will be distributed to subscribers upon enrollment, at 6 weeks, 3 months, and 6 months. Key informant interview questionnaire will be developed to assess and explore the factors that contribute to stress, burnout, anxiety, and depression among teachers, the impact of the Wellness4Teachers program on the levels of these mental health variables in subscribers, and subscriber satisfaction with the daily supportive text messaging program.

### Ethics Approval

The study has approval from the University of Alberta Ethics Review Board (Pro00117558) and is currently seeking approval from the Dalhousie University Human Research Ethics Review Board. Consent to participate will be implied when participants complete and submit the web-based survey responses.

### Study Setting

The study will occur in Alberta and Nova Scotia. Alberta is a province in Western Canada, with an estimated population of 4,067,175 in 2016 [47]. Elementary and high schools in Alberta are run by 61 school boards [48]. In 2013, Alberta’s school jurisdictions employed approximately 35,000 full-time equivalent teachers. Alberta has more than 150 private school authorities, which operate about 180 schools and serve more than 38,000 students [49]. Nova Scotia is a province in Eastern Canada, with a population of 1 million residents in 2021, according to Statistics Canada [50]. Elementary and high schools in Nova Scotia are located in 8 school districts with 7 English-language school boards and 1 French–first language school board. Public schools in Nova Scotia are managed by the provincial department of education, called Education and Early Childhood Development. There were 372 public schools in Nova Scotia in the 2017-18 school year [51,52]. Nova Scotia also has more than 20 private or independent schools, many of which are in Halifax, the provincial capital [53]. There are more than 10,000 public school teachers in Nova Scotia, who are represented by the Nova Scotia Teachers Union [54].

### Outcomes and Measures

Clinical outcomes will be assessed using validated screening scales for self-reported symptoms, including Perceived Stress Scale (PSS-10; a score of ≥10 indicates a likely moderate or high stress) [55], the 7-item GAD scale (GAD-7; a score of ≥10 indicates likely generalized anxiety disorder) [56], the Patient Health Questionnaire-9 (PHQ-9; a score ≥10 indicates likely MDD) [57], and the Brief Resilience Scale (BRS; mean scores ranging from 1.00 to 2.99 indicate low resilience, from 3.00 to 4.30 suggest normal resilience, and from 4.31 to 5.00 suggest high resilience) [58,59].Validated scales have been chosen to better understand self-reported symptoms and potential symptom severity and to screen for the likely presence of psychopathology. These scales are not intended as diagnostic tools but instead are to identify risk factors and early symptoms of potential mental disorders such as depression and anxiety disorders. In addition, we will assess burnout using the Maslach Burnout Inventory (MBI) [60]. The MBI-Educators Survey will be used in this case, which is a version of the original MBI for use with educators, including teachers, administrators, other staff members, and volunteers working in any educational setting [61]. The primary outcome measures will be prevalence of moderate-to-high stress, burnout, likely GAD, likely MDD, and low resilience at baseline in subscribers of Wellness4Teachers. Other primary outcome measures will be changes in prevalence of moderate-to-high stress, burnout, likely GAD, likely MDD, and low resilience as well as changes in mean scores on the PSS-10, MBI, GAD-7, PHQ-9, and the BRS from baseline to 6 weeks, 3 months, and 6 months. Secondary outcome measures will include sociodemographic, clinical, and professional correlates of moderate-to-high stress, burnout, likely MDD, likely GAD, and low resilience among subscribers of Wellness4Teachers at baseline. An exploratory outcome will be a measurement of the adoption of the Wellness4Teachers program by teachers in Alberta and Nova Scotia. This will be assessed by measuring the proportion of the target population (teachers in Alberta and Nova Scotia) who subscribe to the daily supportive text messages.

### Sample Size Estimation

With a teacher population of approximately 45,000 in Alberta and Nova Scotia, using a web-based script [62], we estimate that the sample size needed for our prevalence estimates with a 95% CI and 3% margin of error for moderate-to-high stress, burnout, likely GAD, likely MDD, and low resilience among teachers in Alberta and Nova Scotia is 1043. Based on the response rates achieved for the Text4Hope and Text4Mood programs in Alberta [35,63,64], we expect a maximum of 20% survey completion rate for the Wellness4Teachers program. Thus, to achieve the 1043 completed surveys at baseline, we expect to enroll 5515 teachers on the Wellness4Teachers program within 12 months.

### Statistical Analysis

Quantitative data from the surveys will be analyzed using SPSS (version 25, IBM Corp) [65]. Descriptive statistics will be provided for demographic, clinical, and burnout-related variables based on the province of residence. Cross-tabular analyses using the chi-square test will explore the differences between elementary and high school teachers with respect to demographic, clinical, and professional variables. Descriptive characteristics will be presented as numbers and percentages, and a 2-tailed *P*≤.05 will be used to determine statistical significance for all analyses. We will use the chi-square test and logistic regression analysis to identify demographic, clinical, and work-related correlates of anxiety, depression, stress, low resilience, and burnout for elementary and high school teachers separately. Furthermore, we will assess the impact of the Wellness4Teachers program in reducing moderate-to-high stress, burnout, likely GAD, likely MDD, and improving resilience among Wellness4Teachers subscribers by assessing the mean changes in these parameters from baseline to 6 weeks, 3 months, and 6 months using the 2-tailed paired *t* test. To assess the effects of the intervention against a control group, we will choose a defined period of 3 months during the study period (eg, from the beginning of November 2022 to the end of January 2023) and compare the prevalence and mean scores on standardized scales for stress, burnout, anxiety, and depression at 6 weeks for subscribers who have received the daily supportive text messages for 6 weeks (intervention group) to the baseline prevalence and mean scores on the same scales for new subscribers during the period (control group). Surveys with more than 50% missing responses will be omitted from data analysis. For the included survey responses, there will be no imputation for missing data, and the analysis and results will be based on completed survey data.

We will analyze qualitative data (obtained by audio recording and transcribing responses from key informant interviews) using thematic analysis with NVivo (version 9; QSR International). The results will be reported as themes and subthemes supported by verbatim quotes.

### Hypothesis

We hypothesize that the prevalence of stress, burnout, symptoms of anxiety and depression, as well as low resilience among teachers in Alberta and Nova Scotia would be comparable to those reported in other jurisdictions [8,66].

We also hypothesize that factors such as gender, sex, age, marital status, number of years teaching, grade of teaching, and school type (elementary vs high school) will have an association with burnout and other psychological disorders in teachers [67-72].

Finally, we hypothesize that the Wellness4Teachers program will reduce the prevalence and severity of stress, anxiety, depression, burnout, and low resilience symptoms among teachers by at least 20% [43]. This specific hypothesis is based on related research findings. In the 2 RCTs conducted in Ireland and Canada, there was a greater than 20% reduction in depression symptom scores in the intervention group compared to the control group [73,74]. Furthermore, for the Text4Hope program in Alberta, there was a greater than 20% reduction in anxiety symptom scores in subscribers at 6 weeks and 3 months [36,37].

## Results

The Wellness4Teachers program is expected to be launched in September 2022 when the new academic year is scheduled to begin. Enrollment will last for approximately 1 year, and data collection will continue for another 6 months. Study results will be disseminated with stakeholders in the education sector in Alberta and Nova Scotia and globally through workshops, conference presentations, and peer-reviewed publications.

## Discussion

### Expected Findings

Stress, burnout, anxiety, and depression can have a significant impact on the health, lifestyle, psychological safety, and well-being of teachers, leading to low levels of resilience and reduced professional fulfilment. The psychological impact is likely to be more significant for those with prior mental health conditions or those who have been exposed to previous traumas. Mental health support for groups such as teachers require innovative techniques that can provide support for more teachers. This protocol outlines the use of mobile health technology as a convenient, cost-effective, and accessible means for implementing a psychological intervention for teachers who may be experiencing stress, burnout, anxiety, and depression, and improve their overall resilience.

The outcomes of this study will be evaluated with standardized and empirically validated questionnaires. The findings will contribute to knowledge on eHealth approaches in the education sector and will provide key information about the prevalence rates of stress, burnout, anxiety, depression, and low resilience as well as their correlates in teachers. The findings will also provide evidence of effectiveness for the use of daily supportive text messaging programs to address stress, burnout, anxiety, depression, and low resilience among teachers. Information from this study will thus be critical and useful for informing school policy and decision makers regarding psychological interventions for teachers, especially during the ongoing COVID-19 pandemic or similar stressful situations. We hope that the outcome of this study will promote the integration of supportive text messaging into many organizations’ occupational health programs to provide readily available psychological support to individuals who need it, while at the same time improving their overall resilience and promoting professional fulfilment.

### Limitations of the Study

First, the self-report scales used to assess mental health variables such as likely MDD, although standardized, are not meant to be diagnostic. Second, it is possible that participants’ demographics in the study may not reflect the demographics of the teachers’ population in Alberta or Nova Scotia, and therefore the study findings may not be generalizable to all teachers in the 2 provinces. Furthermore, web-based surveys with survey links delivered through text messages usually achieve a response rate of less than 20% [42,63,64,75-78], and therefore it is possible that we may not achieve our desired sample size. Lastly, the supportive text messages will be delivered for 6 months, and the outcome measures will be evaluated at 6 weeks, 3 months, and the end point of 6 months. It is unclear what the effects of the intervention would be if it were prolonged. It is also unclear if the benefits of the intervention would wane with the cessation of the daily supportive text messages. These limitations notwithstanding, this study is the first to examine the prevalence and correlates of stress, burnout, anxiety, and depression among teachers in the provinces of Alberta and Nova Scotia in Canada, aided by a text message program. This study is also the first globally to assess if daily supportive text messages delivered through the Wellness4Teachers program can reduce the prevalence and severity of stress, burnout, anxiety, and depression, and improve resilience among elementary and high school teachers. The findings would therefore be of interest to policy makers, especially those working in the education sector.

### Conclusion

The outcome of this study will establish the prevalence and correlates of the common risk factors for psychological disorders under study in teachers in Alberta and Nova Scotia. The study will have a significant impact on the management of stress, burnout, anxiety, depression, and low resilience among teachers. If the findings are positive, the Wellness4Teachers program can be promoted as a tool to support the mental health of teachers in Canada and internationally. The study outcome will also compliment policy decision-making for health care resource allocation in support of the education sector.

## Abbreviations

BDI: Beck Depression Inventory
BRS: Brief Resilience Scale
GAD: generalized anxiety disorder
MBI: Maslach Burnout Inventory
MDD: major depressive disorder
PHQ-9: Patient Health Questionnaire-9
PSS: Perceived Stress Scale
RCT: randomized controlled trial
